# Maximising mentorship: Variations in laboratory mentorship models implemented in Zimbabwe

**DOI:** 10.4102/ajlm.v3i2.241

**Published:** 2014-11-03

**Authors:** Phoebe Nzombe, Elizabeth T. Luman, Edwin Shumba, Douglas Mangwanya, Raiva Simbi, Peter H. Kilmarx, Sibongile N. Zimuto

**Affiliations:** 1 Zimbabwe National Quality Assurance Programme (ZINQAP) Trust, Zimbabwe; 2 US Centers for Disease Control and Prevention (CDC), United States; 3 Ministry of Health and Child Welfare, Zimbabwe; 4 US Centers for Disease Control and Prevention (CDC), Zimbabwe

## Abstract

**Background:**

Laboratory mentorship has proven to be an effective tool in building capacity and assisting laboratories in establishing quality management systems. The Zimbabwean Ministry of Health and Child Welfare implemented four mentorship models in 19 laboratories in conjunction with the Strengthening Laboratory Management Toward Accreditation (SLMTA) programme.

**Objectives:**

This study outlines how the different models were implemented, cost involved per model and results achieved.

**Methods:**

Eleven of the laboratories had been trained previously in SLMTA (Cohort I). They were assigned to one of three mentorship models based on programmatic considerations: Laboratory Manager Mentorship (Model 1, four laboratories); One Week per Month Mentorship (Model 2, four laboratories); and Cyclical Embedded Mentorship (Model 3, three laboratories). The remaining eight laboratories (Cohort II) were enrolled in Cyclical Embedded Mentorship incorporated with SLMTA training (Model 4). Progress was evaluated using a standardised audit checklist.

**Results:**

At SLMTA baseline, Model 1–3 laboratories had a median score of 30%. After SLMTA, at mentorship baseline, they had a median score of 54%. At the post-mentorship audit they reached a median score of 75%. Each of the three mentorship models for Cohort I had similar median improvements from pre- to post-mentorship (17 percentage points for Model 1, 23 for Model 2 and 25 for Model 3; *p* > 0.10 for each comparison). The eight Model 4 laboratories had a median baseline score of 24%; after mentorship, their median score increased to 63%. Median improvements from pre-SLMTA to post-mentorship were similar for all four models.

**Conclusion:**

Several mentorship models can be considered by countries depending on the available resources for their accreditation implementation plan.

## Introduction

Since its inception in 1980, the Zimbabwean Ministry of Health and Child Welfare (MoHCW) has been working to develop and manage the country’s healthcare delivery system, which faces challenges common to other healthcare systems in sub-Saharan Africa.^1^ Zimbabwe’s situation was worsened by the effects of an economic crisis,^2^ which resulted in the exodus of highly-skilled health professionals to more stable countries, reduced funding for healthcare programmes and an inconsistent supply of basic resources.^3^ The Zimbabwe National Quality Assurance Programme (ZINQAP) is a non-profit organisation established in 1994 with a mandate to assist medical laboratories to attain and maintain a high standard of quality in their work. Because high-grade laboratory services are the cornerstone of a well-functioning health delivery system, in 2010 the MoHCW earmarked laboratory services for strengthening, developed a National Laboratory Strategic Plan and embarked on implementation of Quality Management Systems (QMS). A cooperative agreement was signed between ZINQAP and the US Centers for Disease Control and Prevention (CDC) to assist the MoHCW in the expansion and strengthening of laboratory technical capacity and quality nationwide.

The Strengthening Laboratory Management Toward Accreditation (SLMTA) programme was launched in 2009 by CDC and the World Health Organization’s Regional Office for Africa (WHO AFRO) as a training curriculum for achieving immediate, measurable improvement in laboratories in resource-limited settings, using available resources.^4^ MoHCW and ZINQAP adopted SLMTA as the tool to establish QMS in Zimbabwean laboratories.

In January 2010, ZINQAP conducted baseline audits in 20 laboratories which included all the reference, central and provincial laboratories as well as some private laboratories. From these 20 laboratories, 11 were selected for the initial SLMTA cohort, based on their level in the tiered laboratory system, baseline audit scores, staff availability and geographic location. These 11 laboratories included reference (*n* = 2), central hospital (*n* = 4), private (*n* = 2), provincial (*n* = 2) and city council (*n* = 1) laboratories. The Laboratory Manager and the Quality Officer from each of the 11 laboratories were trained in the SLMTA three-workshop series. Improvement projects were assigned to be completed in their laboratories following each workshop, and exit audits were conducted after the final workshop so as to determine the impact of the intervention. The entire process from baseline audit to exit audit lasted 22 months.

Despite marked improvements, laboratory managers believed that they could achieve greater results if they received assistance in setting up their QMS for longer periods of time from a laboratory professional who was more experienced in QMS establishment and implementation. Reports from other countries such as Lesotho and Cameroon suggested that mentorship coupled with the SLMTA programme was an effective tool for achieving improvement in quality systems.^5,6^ The MoHCW thus decided to implement a mentorship programme in the laboratories that had completed the SLMTA programme in an effort to continue improving quality management. Several mentorship models were implemented based on available funds, resources and staff allocation. In March 2012, eight more laboratories were recruited in a second round of SLMTA (six district-level and two provincial); mentorship was incorporated into the programme for this cohort.

This study examines the results achieved by the 19 laboratories after implementing four different mentorship models in order to determine their effectiveness, relative cost and lessons learnt.

## Research methods and design

### Mentor recruitment and training

**FIGURE 1:**
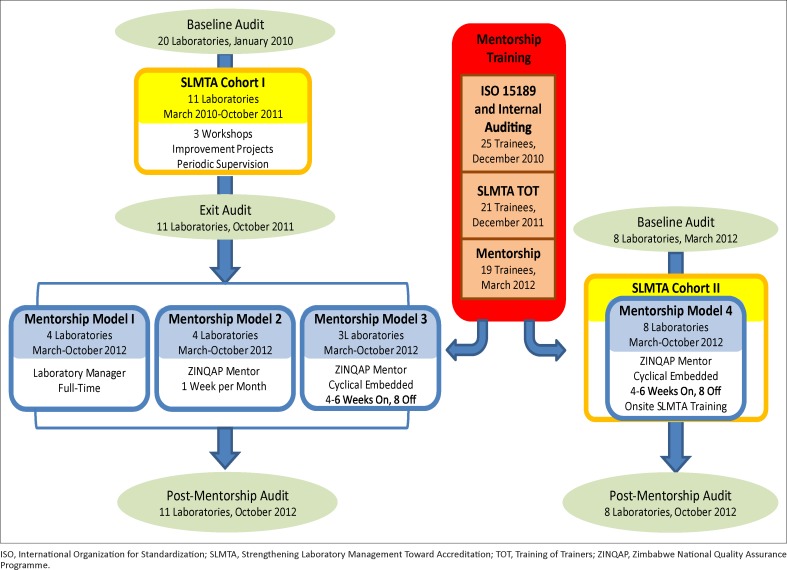
SLMTA and mentorship implementation in Zimbabwe.

ZINQAP established a Training and Mentorship Department, which was mandated to assist medical laboratories in setting up quality systems for improved patient care and to help them work towards accreditation. ZINQAP was responsible for implementing the SLMTA and laboratory mentorship programmes with funding from the US President’s Emergency Plan for AIDS Relief (PEPFAR). The department recruited five local mentors who were qualified Medical Laboratory Scientists with a four-year university degree and who had at least five years’ experience working in a routine medical laboratory as well as some experience in implementing laboratory QMS. A lead mentor, who was a SLMTA Master Trainer, was hired to coordinate the programme and to ensure that the mentors fulfilled their assignments. After recruitment, the mentors attended three training courses designed to equip them with the knowledge and skills needed to mentor laboratories in establishing QMS: International Organization for Standardization (ISO) 15189 Requirements and Internal Auditing; SLMTA Training-of-Trainers; and Mentorship (Figure 1).

### Mentorship models

Laboratories were assigned to one of four mentorship models. Assignment was purposive, based on geographic location, performance of the laboratory managers, laboratory type and timing of SLMTA implementation. Mentorship took place from March to October 2012 for all four models.

#### Model 1: Laboratory manager mentorship after SLMTA

The four Cohort I laboratories with the strongest laboratory managers based on performance during SLMTA implementation were selected for mentorship Model 1. These laboratory managers were trained in the three previously-described training courses. They were then tasked to mentor their own laboratory staff members toward accreditation. ZINQAP mentors were available to assist in the development of work plans and to provide clarification as needed during the scheduled monthly support visits, which lasted at least one full day each month, but were extended if the laboratory manager needed more assistance.

#### Model 2: One week per month mentorship after SLMTA

The remaining private, reference and central laboratories from Cohort I were assigned to mentorship Model 2. One ZINQAP mentor was responsible for assisting these four laboratories and was on site in each laboratory for one week of every month. At the end of each mentorship week staff members were assigned tasks to complete during the three weeks of the mentor’s absence.

#### Model 3: Cyclical embedded mentorship after SLMTA

The three provincial laboratories in Cohort I were each mentored by an embedded ZINQAP mentor who was based at the laboratory for six weeks, away for eight weeks and then back for another four weeks. The mentor assigned tasks for laboratory staff to complete between visits.

#### Model 4: Cyclical embedded mentorship incorporated with SLMTA

The eight laboratories in the second SLMTA cohort had mentorship combined with the SLMTA training. Each mentor from Model 3 above worked with two of the six district-level Model 4 laboratories in their province in a cyclical manner. In Cycle 1, after working with the provincial laboratory from Model 3, these mentors then relocated to the first Model 4 district laboratory for four weeks followed by the second district laboratory for another four weeks. More time was spent at the Model 3 provincial laboratories because they have more sections and staff members than the district laboratories. In Cycle 2, all laboratories were mentored for four weeks each. Two new provincial laboratories were also added in Cohort II. These were mentored by an additional ZINQAP mentor who worked in each laboratory in the same cyclical manner as Model 3 above.

For Cohort II, SLMTA was delivered in a continuous, decentralised format, rather than in workshops. The mentors, who were also SLMTA trainers, conducted the first half of the SLMTA curriculum in Cycle 1 and the second half in Cycle 2. At least one activity was conducted each day and was attended by all laboratory staff, including general and administrative staff, in order to encourage cooperation. All activities were conducted in relation to the work plan. Improvement projects, which emanated from specific activities, were assigned to staff responsible for that scope of work immediately after the daily session. For example, after conducting the ‘What Is Wrong with the Storeroom?’ activity, the stores person was assigned to put the storeroom in order, with the mentor showing them how. Other improvement projects, such as writing of procedures, were conducted as ongoing projects, continuing even after the mentor had left the laboratory.

### Audits

The Cohort I baseline audits were conducted in January 2010 using the initial 2009 version of the WHO AFRO Laboratory Accreditation Checklist. In 2012, the checklist was revised slightly and renamed the WHO AFRO Stepwise Laboratory Quality Improvement Process Towards Accreditation (SLIPTA) checklist,^7^ which was used for all subsequent audits. Based on these audits, a laboratory’s progress is evaluated using a zero to five star rating, whereby 0% – 54% = zero stars, 55% – 64% = one star, 65% – 74% = two stars, 75% – 84% = three stars, 85% – 94% = four stars and 95% – 100% = five stars.

For the 11 laboratories in Cohort I, the SLMTA exit audit conducted in October 2011 was used as the baseline for assessing the impact of mentorship. The eight laboratories in Cohort II had their baseline audits conducted in March 2012. The post-mentorship audit to assess the mentorship programme in all 19 laboratories was conducted in October 2012 by two independent WHO-trained auditors from organisations based in Uganda and Botswana.

### Data analysis

Differences between audit scores were calculated and tested using the Student’s *t*-test with a significance level of *p* = 0.05.

### Cost analysis

A descriptive retrospective analysis evaluated the financial costs (cash expenditures) of implementing each of the four mentorship models. This was done through the use of an inventory of all resources and payments made for the programme.^8^ Costs calculated included: the three training courses which were attended by the mentors; mentorship costs such as salaries, travel and lodging, internet access and laptops; and supervisory costs (Table 1). This analysis was limited to the direct costs of providing mentorship and supervision only. Other components of SLMTA implementation, such as SLMTA workshops, improvement projects and staff time needed to complete the programme were not included, nor was opportunity cost of the reduced time available for regular duties of the laboratory manager in Model 1. Costs were calculated on a per mentor basis, as well as per laboratory, factoring in the number of laboratories assigned to each mentor. Amounts are reported in US dollars, which was the official currency of Zimbabwe at the time of the study.

**TABLE 1:** Cost of implementing mentorship in conjunction with Strengthening Laboratory Medicine Toward Accreditation (SLMTA) programme for four mentorship models in Zimbabwe.

Model	Cost in United States Dollars (USD) per mentor for the eight months under study									Laboratories per mentor	Cost per laboratory		
	Training			Cost of Mentorship							Mentorship	Supervision	Total
	ISO and Internal Auditing	Mentorship	SLMTA Training of Trainers	Internet access	Lodging	Fuel	Salary	Equipment	Total				
Model 1: Laboratory Manager Mentorship after SLMTA	750	613	4123	-	-	-	-	-	**5486**	1	5486	928	**6414**
Model 2: One Week per Month Mentorship after SLMTA	750	613	4123	400	-	256	12 000	900	**19 042**	4	4761	928	**5689**
Model 3: Cyclical Embedded Mentorship after SLMTA	750	613	4123	400	8400	224	12 000	900	**27 410**	3	9137	464	**9601**
Model 4: Cyclical Embedded Mentorship Incorporated with SLMTA	750	613	4123	400	8400	224	12 000	900	**27 410**	3	9137	464	**9601**

**Notes on costs:** ISO and Internal Auditing Training ($750). Training fee $440 (covered facilitator travel, accommodation, and fees). Conference package $35 per day for five days (covered stationary, venue, and refreshments). Participant per diem $15 per day for five days. Participant travel $60 (bus fare at $30 each way). Mentorship Training ($613): Trainer fee $268 (covered facilitator travel, accommodation, and fees). Conference package $35 per day for five days (covered stationary, venue, and refreshments). Materials $10. Copy of the ISO 15189 Standard $25. Participant per diem $15 per day for five days. Participant travel $60 (bus fare at $30 each way). SLMTA Training of Trainers ($4123): Trainer fee $900 (covered facilitator travel, accommodation, and fees). Conference package $60 per day for 11 days. Materials and training equipment $523. Participant accommodation and per diem $180 per day for 11 days. Participant travel $60 (bus fare at $30 each way). Internet Access ($400): $50 per month for eight months. Lodging ($8400): 16 weeks in home area (no lodging). 16 weeks at district laboratories $75 per day (covered lodging and meals). Fuel ($224–$256): Model 2: average distance to mentored laboratories 10 km round trip daily, 22 days per month for eight months = 1760km. At 10 km per litre and $1.45 per litre = $256 per mentor. Model 3 and 4: average distance to mentored laboratories 193 km round trip, four visits to each of two laboratories = 1544 km. At 10 km per litre and $1.45 per litre = $224 per mentor. Salary ($12 000): $1500 per month for eight months. Equipment ($900): Each mentor received a laptop computer $900. Supervision ($464-$928): $58 per visit for travel and per diem. Two visits per month for eight months for Models 1 and 2, one visit per month for Models 3 and 4 due to embedded mentorship.													

ISO, International Organization for Standardization; SLMTA, Strengthening Laboratory Management Toward Accreditation.

## Results

**FIGURE 2:**
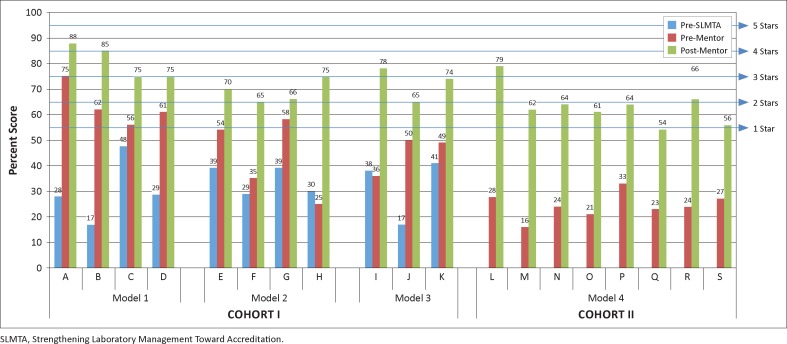
Comparison of results based on four mentorship models implemented in Zimbabwe.

Prior to implementing the SLMTA programme, Cohort I laboratories had a median baseline score of 30% and none of the 11 laboratories had attained scores high enough to reach a one-star rating (Figure 2). After SLMTA implementation, the laboratories improved to reach a median score of 54% and five of the laboratories attained at least one star. After the post-SLMTA mentorship programme the laboratories reached a median score of 75%, with two laboratories at four stars, five laboratories at three stars and four laboratories at two stars. Each of the three mentorship models for Cohort I (Models 1–3) had similar improvements from pre- to post-mentorship, with a median improvement of 17 percentage points for Model 1, 23 percentage points for Model 2 and 25 percentage points for Model 3 (*p* > 0.10 for each comparison).

The eight Cohort II laboratories (Model 4), which received mentorship along with on-site SLMTA training, had a median baseline score of 24%, with no laboratories reaching one star. After SLMTA/mentorship, one of the laboratories reached three stars, three laboratories reached two stars, three laboratories reached one star and the remaining laboratory was just one point away from achieving one star. The median scores of the laboratories increased 39 percentage points to 63%. These improvements were greater than those observed from the Cohort I laboratories (Models 1–3) at their SLMTA exit audit (median improvement 24 percentage points; *p* = 0.01), but similar to Cohort I improvements from pre-SLMTA baseline to post-mentorship (median improvement 53 percentage points for Model 1, 34 percentage points for Model 2 and 40 percentage points for Model 3; *p* > 0.10 for each comparison).

### Cost of the mentorship models

The total cost per mentor for attending the three courses was $5486 (Table 1). The other costs of mentorship varied by model, with total costs per mentor of $5486 for Model 1, $19 042 for Model 2 and $27 410 for Models 3 and 4. After factoring in the number of laboratories assigned per mentor and the supervisory cost, the costs per laboratory ranged from $4761 for Model 2 to $9137 for Models 3 and 4.

## Discussion

Unlike other studies that have examined the combined effect of mentorship and SLMTA,^9,10,11^ the delayed implementation of mentorship in Zimbabwe allowed us to evaluate the incremental impact of mentorship separate from SLMTA training.

Cohort I laboratories registered substantial improvement during SLMTA implementation, from a median baseline of 30% to a median exit audit of 54%. They then improved even further, to a median of 75% after mentorship. Whilst it is not possible with our data to determine what the impact of mentorship would have been without first implementing the SLMTA programme, it is clear from these results that the addition of mentorship had a beneficial effect on the laboratories over and above the effect of SLMTA training alone. Cohort II, which implemented mentorship alongside SLMTA training, saw a similar total improvement, from 24% to 63%, suggesting that whether performed sequentially or simultaneously, mentorship can play an effective role in laboratory quality improvement.

One laboratory in Cohort I had regressed from SLMTA baseline to exit score; the two SLMTA participants reported that they were unsuccessful in getting the other laboratory personnel to work as a team in order to implement the improvement projects. After mentorship, their laboratory’s audit score more than doubled; mentorship was successful because all laboratory staff were mentored on site, so they all understood the process and were eager to play their part.

The mentorship models showed similar improvements of approximately 17–25 percentage points for mentorship only and 39 percentage points for mentorship combined with SLMTA. We were not able to conclude that one model was better than the others – in part because of the small sample sizes, which limited statistical power, but also because laboratories were not assigned randomly to mentorship models but were selected based on the judgement of the programme leadership team. Characteristics of the laboratories that were used to assign mentorship model (such as geographic location and effectiveness of laboratory managers) may also have affected laboratory performance. However, there were important lessons learned about the advantages and disadvantages of each model, as well as the situations in which they should be used (Table 2).

**TABLE 2:** Implementation of mentorship models in conjunction with the Strengthening Laboratory Management Toward Accreditation (SLMTA) programme in Zimbabwe.

Cohort	Model	Number of laboratories	Laboratory types	Rotation	Median Audit Scores (%)			Criteria						Recommended use
					Baseline	Exit	Post-mentorship	Continuous mentorship	Dedicated mentor	Builds local mentorship capacity	Inexpensive	Frequent visits	Longer visits	
Cohort I	Model 1: Laboratory Manager Mentorship after SLMTA	4	Private, reference and central	Continuous	29	62	80	✓	✓	✓	✓	✓	✓	Laboratories with strong managers
	Model 2: One Week per Month Mentorship after SLMTA	4	Private, reference and central	1 week on, 3 weeks off	35	44	68	-	✓	-	✓	✓	-	Laboratories in close proximity to a qualified mentor
	Model 3: Cyclical Embedded Mentorship after SLMTA	3	Provincial	6 weeks on, 8 weeks off, 4 weeks on	38	49	74	-	✓	-	-	-	✓	Remote laboratories and those needing long-term intensive mentorship
Cohort II	Model 4: Cyclical Embedded Mentorship Incorporated with SLMTA	8	Provincial and district	4 weeks on, 4 weeks off, 4 weeks on	24	63	63	-	✓	-	-	-	✓	Remote laboratories and those needing long-term intensive mentorship

SLMTA, Strengthening Laboratory Management Toward Accreditation.

### Model 1: Laboratory manager mentorship after SLMTA

This model, which utilised existing laboratory managers as mentors, was targeted to laboratories which had excelled in SLMTA implementation and whose laboratory managers were strong and committed to implementing a QMS. Because of their established authoritative role as laboratory managers, these mentors were able to enforce the new quality culture in the laboratory. Also, because they were full-time laboratory employees, they were able to provide mentorship on a continuous basis throughout the programme. Perhaps the greatest advantage of this model is that it builds capacity at the grass-roots level, as these laboratories will benefit from having trained mentor–managers long after the programme has ended. On the other hand, these mentors had simultaneous management duties, including analysing patient results, attending hospital management meetings, representing the laboratory at regional and national meetings and authorising QMS documents. Whilst some of these duties could be delegated to deputies, these mentors were not able to devote all of their time to mentorship. This model may be best suited for situations in which there are strong laboratory managers who are supportive of setting up a QMS and who can handle both mentorship and administrative duties.

### Model 2: One week per month mentorship after SLMTA

This model was implemented in four laboratories which were located within a 10 km radius of the mentor’s residence. The mentor worked with each laboratory for one week at a time, assigning tasks to be completed in the three weeks he was away. One benefit of this model is that it utilises full-time professional mentors, allowing them to focus completely on mentorship duties. Also, laboratories have mentorship guidance each month, ensuring continuous progress. This was the least expensive mentorship model. This model is recommended for laboratories which are in close proximity to a qualified mentor who can commute daily to the laboratories.

### Model 3: Cyclical embedded mentorship after SLMTA

The laboratories enrolled under this model were located in different provinces, requiring extensive travel for mentors. The main benefit of this model was that the laboratories had full-time mentors on site for long periods of time, allowing in-depth training and oversight of major improvement projects. However, they were also without their mentors for eight weeks between visits; problems arising may not have been identified and corrected for as long as two months. Also, travel and lodging expenses were relatively expensive, as the greater distances required mentors to travel and lodge on site. This model is most appropriate for more remote laboratories needing long-term intensive mentorship in order to make substantive changes.

### Model 4: Cyclical embedded mentorship incorporated with SLMTA

For our study, it was not possible to separate the effects of mentorship and of SLMTA training for Model 4, as they were conducted simultaneously. Standard SLMTA implementation includes mentorship between training sessions.^4^ Several countries have utilised the embedded mentorship model incorporated with SLMTA training, with good results.^11,12,13,14^ Our SLMTA training for Cohort II was conducted in a decentralised model rather than in centralised workshops; one study in Cameroon also found decentralised training to be effective.^12^

In general, the strengths and weaknesses of Model 4 are the same as those discussed for Model 3. In addition, there are benefits associated with conducting concurrent mentorship and SLMTA training. Logistically, it is easier to coordinate a single united programme, with everyone’s focus on laboratory quality. Also, mentors worked through issues discussed at the previous training session, immediately reinforcing lessons learned in the classroom and assisting in translating those lessons to real-world problems in the laboratory. And because mentors are also trainers, there may be some cost sharing between the training and mentorship components of the programme, which was not evaluated in this study.

### Limitations of the study

This was a descriptive study based on a programmatic activity and, as such, is subject to several scientific limitations. Firstly, the small number of laboratories in each mentorship model yielded low power for statistical testing, so the findings from the 19 study laboratories cannot be generalised to the > 200 laboratories nationwide. In addition, both absolute and relative costs will vary by setting based on contextual factors. For example, if the cost of mentor salaries or travel were substantially higher than in our programme, then Model 1, which uses existing laboratory managers as mentors, could prove to be the least expensive option. Secondly, representative sampling and randomised assignment to mentorship models were not done; laboratories were selected purposively based on convenience, programmatic needs and results of baseline audits. Thirdly, Model 4 results cannot be easily compared with the other three models. In our implementation, Model 4 was used for the second cohort of SLMTA; these district and provincial laboratories had substantially lower baseline scores than did those in Cohort I, which were predominantly central and referral laboratories. Thus there was more room for improvement, as laboratories with lower baseline scores tend to have greater score increases than those with higher baseline scores.^15^ In addition, Cohort II laboratories were generally smaller, with fewer departments and staff members and offering a narrower menu of tests. These factors are advantageous in implementing QMS because the mentor has fewer departments to work with; some tasks, such as writing standard operating procedures, are less complicated; and lower staff numbers promote easier team building. Furthermore, lessons learned from the first cohort of SLMTA were incorporated into the second cohort, giving an additional advantage. On the other hand, the laboratories in Cohort I had 34 months over which to make improvements, whilst those in Cohort II were measured after only seven months.

### Conclusion

Based on our findings, we would recommend that countries include mentorship when implementing SLMTA for laboratory quality improvement. Countries should carefully consider which mentorship model or models would be best suited to their individual situation.

## References

[1] Habte D, Dussault G, Dovlo D Challenges confronting the health workforce in sub-Saharan Africa. World Hosp Health Serv. 2004;40(2):23–26, 40–41. 15338994

[2] Kapp C World report: Zimbabwe’s humanitarian crisis worsens. Lancet. 2009;373(9662):447 http://dx.doi.org/10.1016/S0140-6736(09)60151-3 19205080 10.1016/s0140-6736(09)60151-3

[3] Truscott R Zimbabwe’s health challenges. BMJ. 2009;338:b930 http://dx.doi.org/10.1136/bmj.b930 19289414 10.1136/bmj.b930

[4] Yao K, McKinney B, Murphy A, et al Improving quality management systems of laboratories in developing countries: An innovative training approach to accelerate laboratory accreditation. Am J Clin Pathol. 2010;134(3):401–409. http://dx.doi.org/10.1309/AJCPNBBL53FWUIQJ 20716796 10.1309/AJCPNBBL53FWUIQJ

[5] Maruta T, Rotz P, Trevor P Setting up a structured laboratory mentoring programme. Afr J Lab Med. 2013;2(1), Art. #77, 7 pages. http://dx.doi.org/10.4102/ajlm.v2i1.77 10.4102/ajlm.v2i1.77 PMC5637775 29043168

[6] Maruta T, Motebang D, Mathabo L, et al Impact of mentorship on WHO-AFRO Strengthening Laboratory Quality Improvement Process Towards Accreditation (SLIPTA). Afr J Lab Med. 2012;1(1), Art. #6, 8 pages. http://dx.doi.org/10.4102/ajlm.v1i1.6 10.4102/ajlm.v1i1.6 PMC5644515 29062726

[7] World Health Organization’s Regional Office for Africa WHO guide for the stepwise laboratory improvement process towards accreditation in the African region (with checklist) [document on the Internet]. c2012 [cited 2014 September 21]. Available from: http://www.afro.who.int/en/clusters-a-programmes/hss/blood-safety-laboratories-a-health-technology/blt-highlights/3859-who-guide-for-the-stepwise-laboratory-improvement-process-towards-accreditation-in-the-african-region-with-checklist.html

[8] Shumba E, Nzombe P, Mbinda A, et al Weighing the costs: Implementing the SLMTA programme in Zimbabwe using internal versus external facilitators. Afr J Lab Med. 2014;3(2), Art. #248, 6 pages. http://dx.doi.org/10.4102/ajlm.v3i2.248 10.4102/ajlm.v3i2.248 PMC5637799 29043197

[9] Makokha EP, Mwalili S, Basiye FL, et al Using standard and institutional mentorship models to implement SLMTA in Kenya. Afr J Lab Med. 2014;3(2), Art. #220, 8 pages. http://dx.doi.org/10.4102/ajlm.v3i2.220 10.4102/ajlm.v3i2.220 PMC5637804 29043191

[10] Mokobela KO, Moatshe MT, Modukanele M Accelerating the spread of laboratory quality improvement efforts in Botswana. Afr J Lab Med. 2014;3(2), Art. #207, 6 pages. http://dx.doi.org/10.4102/ajlm.v3i2.207 10.4102/ajlm.v3i2.207 PMC5637812 29043187

[11] Audu RA, Onubogu CC, Nwokoye NN, et al Improving quality in national reference laboratories: The role of SLMTA and mentorship. Afr J Lab Med. 2014;3(2), Art. #200, 7 pages. http://dx.doi.org/10.4102/ajlm.v3i2.200 10.4102/ajlm.v3i2.200 PMC5637787 29043183

[12] Ndasi J, Dimite L, Mbome V, et al Decentralised facility-based training as an alternative model for SLMTA implementation: The Cameroon experience. Afr J Lab Med. 2014;3(2), Art. #231, 6 pages. http://dx.doi.org/10.4102/ajlm.v3i2.231 10.4102/ajlm.v3i2.231 PMC5637810 29043194

[13] Nkwawir SC, Batumani NN, Maruta T, Awasom CN From grass to grace: How SLMTA revolutionised the Bamenda Regional Hospital Laboratory in Cameroon. Afr J Lab Med. 2014;3(2), Art. #203, 6 pages. http://dx.doi.org/10.4102/ajlm.v3i2.203 10.4102/ajlm.v3i2.203 PMC5637803 29043186

[14] Guevara G, Gordon F, Irving Y, et al The impact of SLMTA in improving laboratory quality systems in the Caribbean Region. Afr J Lab Med. 2014;3(2), Art. #199, 9 pages. http://dx.doi.org/10.4102/ajlm.v3i2.199 10.4102/ajlm.v3i2.199 PMC4826060 27066396

[15] Yao K, Luman ET, SLMTA Collaborating Authors Evidence from 617 laboratories in 47 countries for SLMTA-driven improvement in quality management systems. Afr J Lab Med. 2014;3(2), Art. #262, 11 pages. http://dx.doi.org/10.4102/ajlm.v3i2.262 10.4102/ajlm.v3i2.262 PMC4706175 26753132

